# Emerging Roles of Exosomal Circular RNAs in Cancer

**DOI:** 10.3389/fcell.2020.568366

**Published:** 2020-10-08

**Authors:** Takahiro Seimiya, Motoyuki Otsuka, Takuma Iwata, Chikako Shibata, Eri Tanaka, Tatsunori Suzuki, Kazuhiko Koike

**Affiliations:** Department of Gastroenterology, Graduate School of Medicine, The University of Tokyo, Tokyo, Japan

**Keywords:** circular RNA, circRNA, exosome, exosomal circRNA, cancer progression, biomarker

## Abstract

Circular RNA (circRNA) is a type of non-coding RNA that forms a covalently closed continuous loop. The expression pattern of circRNA varies among cell types and tissues, and many circRNAs are aberrantly expressed in various cancers. Aberrantly expressed circRNAs have been shown to play crucial roles in carcinogenesis, functioning as microRNA sponges or new templates for protein translation. Recent research has shown that circRNAs are enriched in exosomes. Exosomes are secretory vesicles that mediate intercellular communication through the delivery of cargo, including proteins, lipids, DNA, and RNA. Exosome-mediated crosstalk between cancer cells and the tumor microenvironment promotes the epithelial-mesenchymal transition, angiogenesis, and immune escape, and thus may contribute to cancer invasion and metastasis. In this review, we discuss the biological functions of exosomal circRNAs and their significance in cancer progression. Additionally, we discuss the potential clinical applications of exosomal circRNAs as biomarkers and in cancer therapy.

## Introduction

Exosomes are secreted vesicles 30–100 nm in diameter that contain host cell-derived cargo, including proteins, lipids, DNA, and RNA. Exosomes deliver their cargo to recipient cells and mediate intercellular communication. Tumor cells communicate with surrounding cells, including other tumor cells, normal cells, fibroblasts, endothelial cells, and immune cells, via exosomes, which helps to form the tumor microenvironment, facilitating proliferation, invasion, and immune escape (Kalluri, 2016; Maia et al., 2018; Wortzel et al., 2019). Furthermore, exosomes derived from tumor cells travel to metastatic sites far from the primary site and create a metastatic niche for colonization by tumor cells (Wortzel et al., 2019). Thus, exosomes play important roles in cancer progression.

Circular RNA (circRNA) is a type of non-coding RNA that forms a covalently closed continuous loop through a process called “back-splicing,” in which a downstream splice donor site is joined with an upstream splice acceptor site. RNA with circular structures were first documented in the 1970s, but were thought to be specific to pathogens such as hepatitis D virus (Kos et al., 1986) and a plant viroid (Sanger et al., 1976). In the 1990s, the presence of several circRNAs was reported (Nigro et al., 1991; Cocquerelle et al., 1992, 1993; Capel et al., 1993), but the overall picture of circRNAs in the human transcriptome remained unclear. Recent advances in RNA sequencing technology and bioinformatics tools have revealed that more than 180,000 circRNAs are present in human transcriptomes (Dong et al., 2018) and the expression pattern of circRNAs varies among cell types and developmental stages (Memczak et al., 2013; Salzman et al., 2013). Furthermore, the abundance of circRNAs is not correlated with that of the cognate linear RNA (Salzman et al., 2013), indicating distinct regulatory mechanisms and cellular functions of circRNAs.

The abundance of circRNA is negatively correlated with cell proliferation rates (Bachmayr-Heyda et al., 2015) and the global expression level of circRNA is downregulated in a diverse range of cancers, including osteosarcoma, colorectal adenocarcinoma, renal cell carcinoma, hepatocellular carcinoma (HCC), lung adenocarcinoma, gastric adenocarcinoma, and prostate cancer (Vo et al., 2019). This is possibly because circRNAs are more stable than linear RNAs (Li et al., 2015). That is, circRNAs can accumulate in non-proliferating cells, whereas in proliferating cells circRNAs are distributed to daughter cells and thus diluted. Despite the global downregulation of circRNA in cancer, some circRNAs are aberrantly expressed and essential for cancer cell proliferation. RNA interference screening using short hairpin RNAs that target back-splicing junctions and linear transcripts outside of circRNA exons, to assess distinct functions of circRNAs and their parental linear RNAs in prostate cancer cells, revealed that 171 circRNAs were essential for proliferation, whereas their linear counterparts were not (Chen S. et al., 2019). These results show that circRNAs are not mere byproducts of splicing, but play important roles in carcinogenesis independent of their linear transcripts.

Recent studies have revealed that circRNAs contribute to carcinogenesis by acting as microRNA (miRNA) sponges, protein scaffolds, or templates for protein translation. Many circRNAs, including circCCDC66, circHIPK3, and circPVT1, have been reported to act as miRNA sponges in cancer (Kristensen et al., 2018, 2019; Chen, 2020). Interestingly, circCCDC66 promotes expression of the oncogene MYC through binding to multiple different miRNAs rather than one particular miRNA (Hsiao et al., 2017). CDR1-AS increases cell surface PD-L1 levels through miR-7-independent mechanisms (Tanaka et al., 2019). circPOK directly binds to interleukin enhancer-binding factor 2/3 (ILF2/3) complex and functions as a coactivator of the ILF2/3 complex, thus regulating pro-proliferative and pro-angiogenic factors (Guarnerio et al., 2019). circACC1 is another circRNA that functions as a protein complex component through direct binding. circACC1 assembles and stabilizes the AMPK complex and mediates metabolic reprogramming in cancer cells (Li et al., 2019b). Human papillomavirus-derived circRNA acts as a template for oncogenic protein translation, resulting in host cell transformation (Zhao J. et al., 2019). Thus, circRNAs in tumor cells and oncogenic viruses promote cancer progression in diverse ways.

Recently, circRNAs were found to be localized to exosomes, stably present over time, and to have biological functions. Ongoing investigations have revealed the roles of exosomal circRNAs in carcinogenesis. In this review, we discuss the biological functions of exosomal circRNAs, their significance in cancer progression, and the clinical applications of exosomal circRNAs.

## circRNA Is Enriched and Stable in Exosomes

The abundance and stability of exosomal circRNAs was first reported based on the RNA-Seq analysis of ribosomal RNA-depleted total RNA from MHCC-LM3 liver cancer cells and cell-derived exosomes (Li et al., 2015). RNA-Seq analysis revealed that the ratio of circRNA levels to their linear counterpart RNA levels in exosomes was about 6-fold higher than that in producer cells. Furthermore, the expression levels of circRNAs were barely altered after incubation at room temperature for up to 24 h (Li et al., 2015). These results indicate that circRNAs are highly enriched and stable in exosomes.

The expression profile of exosomal circRNA is different from that of producer cells (Li et al., 2015; Dou et al., 2016), suggesting that circRNAs are actively incorporated into exosomes. Multiple mechanisms have been implicated in RNA sorting into exosomes, including specific RNA sequences and/or secondary structures associated with RNA binding proteins (O’Brien et al., 2020; Wang M. et al., 2020). However, the precise mechanisms through which circRNAs are sorted into exosomes are not yet clear.

The stability of circRNA may contribute to the enrichment of circRNA in exosomes. circRNA is resistant to exonuclease digestion because it does not have a 5′ or 3′ end (Suzuki et al., 2006), which is responsible for its longer half-life compared to linear RNA (Jeck et al., 2013; Enuka et al., 2016). Because of its long half-life, despite the low efficacy of nascent circRNA synthesis, it accumulates in cells with slow division rates such as neuronal cells (Rybak-Wolf et al., 2015; You et al., 2015; Zhang et al., 2016). Furthermore, an exosome can protect its cargo RNAs from degradation by RNases (Valadi et al., 2007; Skog et al., 2008). Therefore, circRNA that has been incorporated into exosomes can be remarkably stable in exosomes.

The abundance of circRNA in exosomes can be regulated by the miRNA level in producer cells. CDR1-AS, one of the most intensively studied circRNAs, is known to bind to miR-7 and strongly suppress miR-7 activity (Hansen et al., 2013). The level of CDR1-AS is significantly downregulated in exosomes but slightly elevated in cells when miR-7 is ectopically expressed (Li et al., 2015). This finding indicates that sorting of circRNAs into exosomes is regulated, at least in part, by the associated miRNA levels in producer cells.

## Biological Functions of Exosomal circRNAs

**FIGURE 1:**
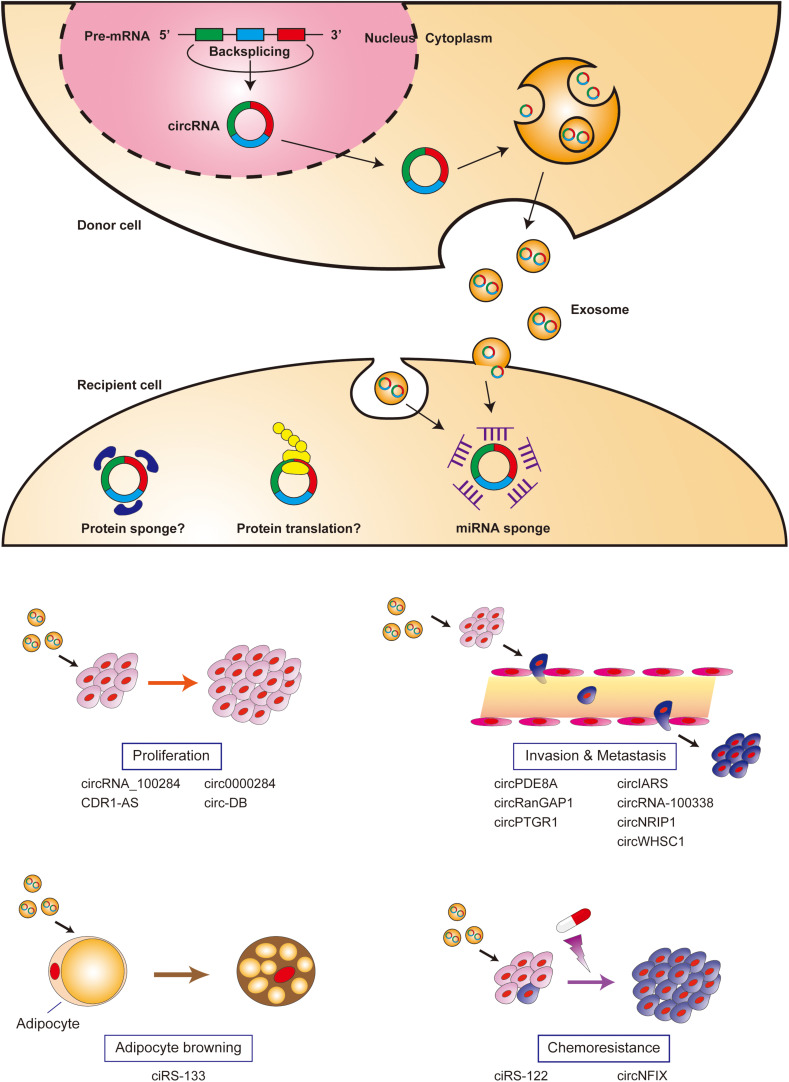
Biological functions of exosomal circRNAs. CircRNAs are generated by back-splicing, packaged into exosomes, and released into the extracellular space. Exosomal circRNAs are internalized into recipient cells via direct fusion with the plasma membrane or endocytosis, and carry out various biological functions in recipient cells.

circRNA retains miRNA sponging activity in the exosome and can modulate miRNA target gene expression in recipient cells. CDR1-AS is the first circRNA reported to retain biological function in the exosome; exosomal CDR1-AS can abrogate miR-7-induced growth suppression in recipient cells by sponging miR-7 (Li et al., 2015). There is growing evidence that exosomal circRNAs are involved in cell proliferation, invasion, the epithelial-mesenchymal transition (EMT), metastasis, chemoresistance, and cancer cachexia through intercellular communication between tumor cells, endothelial cells, and adipocytes (Figure 1 and Table 1).

**TABLE 1:** Biological functions of exosomal circRNAs in cancers.

Exosomal circRNA	Producer cell	Expression	Target miRNA (Target gene)	Function	References
circRNA_100284	Arsenite-transformed hepatic epithelial cell	Upregulated	miR-271 (EZH2)	Accelerates cell cycle and promotes cell proliferation	Dai et al., 2018
CDR1-AS	Hepatocellular carcinoma	Upregulated	miR-1270 (AFP)	Accelerates cell proliferation and migration	Su et al., 2019
circ0000284	Cholangiocarcinoma	Upregulated	miR-637 (LY6E)	Accelerates proliferation and migration	Wang J. et al., 2019
circ-DB	Adipocyte	Upregulated	miR-34a (USP7, CCNA2)	Promotes HCC proliferation and decreases DNA damage	Zhang et al., 2019a
circ0051443	Normal hepatic cells	Downregulated	miR-331-3p (BAK1)	Causes tumor cell apoptosis and cell cycle arrest	Chen W. et al., 2020
circPDE8A	Pancreatic cancer	Upregulated	miR-338 (MACC1)	Promotes EMT Prognostic biomarker	Li Z. et al., 2018
circRanGAP1	Gastric cancer	Upregulated	miR-877-3p (VEGFA)	Promotes migration and invasion	Lu et al., 2020
circPTGR1	Hepatocellular carcinoma	Upregulated	miR-339a (MET)	Promotes migration and invasion	Wang G. et al., 2019
circIARS	Pancreatic cancer	Upregulated	miR-122 (RhoA)	Upregulates the permeability of HUVEC cells	Li J. et al., 2018
circRNA-100338	Hepatocellular carcinoma	Upregulated	Unknown (directly bind to NOVA2)	Upregulates the permeability of HUVEC cells	Huang et al., 2020
circPRMT5	Urothelial carcinoma of the bladder	Upregulated	miR-30c (SNAIL1)	Biomarker for metastasis	Chen et al., 2018
circNRIP1	Gastric cancer	Upregulated	miR-149-5p (AKT1)	Promotes metastasis	Zhang et al., 2019
circWHSC1	Ovarian cancer	Upregulated	miR145, miR1182 (MUC1, TERT)	Promotes peritoneal dissemination	Zong et al., 2019
ciRS-122	Colorectal cancer	Upregulated	miR-122 (PKM2)	Induces chemoresistance	Wang Y. et al., 2020
ciRS-133	Gastric cancer	Upregulated	miR-133 (PRDM16)	Adipocyte browning	Zhang et al., 2019
circ-0051443	Hepatocellular carcinoma	Downregulated	miR-331-3p (BAK1)	Diagnostic biomarker (AUC 0.8089)	Chen W. et al., 2020
hsa_circ_0065149	Gastric cancer	Downregulated	Unknown	Diagnostic biomarker (AUC 0.64)	Shao et al., 2020
circ-KIAA1244	Gastric cancer	Downregulated	Unknown	Diagnostic and prognostic biomarker (AUC 0.7481)	Tang et al., 2018
circ_0000419	Gastric cancer	Downregulated	Unknown	Diagnostic biomarker (AUC 0.84)	Tao et al., 2020
hsa_circ_0001946 and hsa_circ_0043603	Esophageal squamous cell cancer	Downregulated	Unknown	Diagnostic biomarker (Sensitivity: 84%, Specificity: 98%)	Fan et al., 2019
hsa-circ-0004771	Colorectal cancer	Upregulated	Unknown	Diagnostic biomarker (AUC 0.86–0.88)	Pan et al., 2019
hsa_circ_0014235 and hsa_circ_0025580	Lung squamous cell carcinoma	Upregulated	Unknown	Diagnostic biomarker (AUC 0.8)	Wang Y. et al., 2020
hsa_circRNA_0056616	Lung adenocarcinoma	Downregulated	Unknown	Diagnostic biomarker for lymph node metastasis (AUC 0.812)	He et al., 2020
FECR1	Small cell lung carcinoma	Upregulated	miR-584 (ROCK1)	Biomarker for prognosis and response to chemotherapy	Li et al., 2019
circPDAC	Pancreatic cancer	Upregulated	Unknown	Diagnostic biomarker (Sensitivity: 0.45, Specificity: 0.90)	Seimiya et al., 2020
CDR1-AS	Ovarian cancer	Downregulated	miR-1270 (SCAI)	Biomarker for cisplatin resistance	Zhao Z. et al., 2019
circNFIX	Glioma	Upregulated	miR-132 (ABCG2)	Induces chemoresistance Diagnostic biomarker (0.885) and prognostic biomarker	Ding et al., 2020
circ_0044516	Prostate cancer	Upregulated	miR-29a-3p (Unknown)	Diagnostic biomarker (AUC not shown)	Li et al., 2020
circFNDC3B	Papillary thyroid cancer	Upregulated	miR-1178 (TLR4)	Diagnostic biomarker (AUC not shown)	Wu et al., 2020
Other circRNAs determined by bioinformatic analysis	Breast cancer, endometrial cancer, pancreatic ductal adenocarcinoma, lung adenocarcinoma, papillary thyroid carcinoma	Upregulated and downregulated		Diagnostic biomarker (AUC not shown)	Xu et al., 2018; Li et al., 2019a; Wang J. et al., 2019; Yang et al., 2019; Chen F. et al., 2020

### Cell Proliferation

Many exosomal circRNAs have been reported to promote cell proliferation. circRNA_100284 is secreted by arsenite-transformed human hepatic epithelial cells and transferred into surrounding normal cells via exosomes. Exosomal circRNA_100284 accelerates the cell cycle and promotes cell proliferation by acting as a sponge of miR-271 and thereby upregulating EZH2 (Dai et al., 2018). Exosomal CDR1-AS is a circRNA that promotes HCC progression. In HCC cells, upregulated CDR1-AS accelerates cell proliferation and migration by acting as a sponge for miR-1270, promoting the expression of alpha-fetoprotein. CDR1-AS is secreted into exosomes and transferred into surrounding cells, promoting proliferation and migration (Su et al., 2019). circ0000284 is expressed in cholangiocarcinoma cell lines, tumor tissues, and plasma exosomes. In cells, circ00000284 acts as a sponge of miR-637 to upregulate the expression of LY6E and stimulate proliferation, migration, and invasion by cholangiocarcinoma cells. circ0000284 is secreted into exosomes and transferred into surrounding cells, facilitating proliferation and migration (Wang S. et al., 2019). Notably, not only tumor-derived exosomal circRNAs, but also adipocyte-derived exosomal circRNAs, promote tumor growth. Circ-DB is upregulated in serum exosomes obtained from HCC patients with higher body fat ratios, and is highly expressed in exosomes from adipocytes. Circ-DB promotes HCC proliferation and decreases DNA damage through the sponging of miR-34a, thereby promoting USP7 and Cyclin A2 expression (Zhang et al., 2019a).

In contrast to tumor-derived exosomal circRNAs, which promote proliferation in recipient cells, normal cell-derived exosomal circRNAs inhibit proliferation in recipient cells. For example, circ0051443, which is downregulated in plasma exosomes of patients with HCC, is secreted by normal hepatic cells and transferred into adjacent HCC cells, causing tumor cell apoptosis and cell cycle arrest through sponging of miR331-3p and upregulation of BAK1 expression (Chen W. et al., 2020).

### EMT, Invasion, and Metastasis

Exosome-mediated intercellular communication is known to promote tumor invasion and metastasis by inducing the EMT, modifying the tumor microenvironment, and forming pro-metastatic niches (Kalluri, 2016; Maia et al., 2018; Wortzel et al., 2019). Recent studies have revealed that circRNAs in exosomes contribute to tumor invasion and metastasis. circPDE8A is secreted from pancreatic cancer cells, and a high expression level of exosomal circPDE8A in patient plasma was associated with a worse prognosis. In pancreatic cancer cells, circPDE8A acts as a miR-338 sponge to activate the MACC/MET/ERK or AKT pathways and promote invasive growth. Moreover, exosomal circPDE8A promotes the EMT in recipient cells by activating the MACC/MET/ERK or AKT pathway (Li Z. et al., 2018). circRanGAP1 is upregulated in plasma exosomes from preoperative gastric cancer patients compared with healthy control and post-operative gastric cancer patients. More importantly, the plasma exosomes derived from these patients caused enhanced migration and invasion of gastric cancer cells, indicating a potential role for exosomal circRanGAP1 in gastric cancer progression (Lu et al., 2020). circPTGR1 is more abundant in exosomes derived from highly metastatic HCC cells compared with non-metastatic or low-metastatic cells. Co-culture with exosomal circPTGR1 enhances the migration and invasion of HCC cells through the miR-449a/MET pathway. Furthermore, the expression level of circPTGR1 in serum exosomes of HCC patients is associated with advanced clinical stage and worse prognosis (Wang G. et al., 2019).

circRNA in exosomes can promote cancer cell invasion not only by making cancer cells more invasive, but also by altering the tumor microenvironment. circIARS is secreted from pancreatic cancer cells and delivered to human umbilical vein cells (HUVEC) via exosomes. Exosomal circIARS upregulates the permeability of HUVEC cells by sponging miR-122 and upregulating RhoA, enabling easier invasion by pancreatic cancer cells (Li J. et al., 2018). Exosomal circRNA-100338, which is upregulated in a highly metastatic HCC cell line, also promotes HUVEC proliferation, angiogenesis, and permeability. RNA pull-down assay revealed that circRNA-100338 can bind RNA-binding proteins including NOVA2, which was reported to regulate vascular development and lumen formation. This suggests that circRNA-100338 may regulate angiogenesis by interacting with NOVA2, but further study is needed to confirm this (Huang et al., 2020).

Several circRNAs have been reported to play roles in metastasis. circPRMT5, which is frequently upregulated in urothelial carcinoma of the bladder (UCB) cells, acts as a miR-30c sponge and regulates the SNAIL1/E-cadherin pathway to promote the EMT in UCB cells. Moreover, circPRMT5 in serum and urinary exosomes was positively correlated with lymph node metastasis and advanced tumor progression (Chen et al., 2018). The expression level of circNRIP1 in serum from gastric cancer patients is significantly higher than that in healthy controls. Notably, mouse tail injection of gastric cancer cells co-cultured with exosomes derived from circNRIP1-overexpressing cells increased lung metastasis. This result indicates that exosomal circNRIP1 enhances the metastatic potential of gastric cancer cells (Zhang et al., 2019). circWHSC1, which is upregulated in ovarian cancer tissues, acts as a sponge of miR-145 and miR-1182 and upregulates MUC1 and hTERT, resulting in increased cell proliferation, migration, and invasion. Exosomal circWHSC1 shifts the morphology of peritoneal mesothelial cells to fibroblast-like and upregulates N-cadherin and MUC1 in peritoneal mesothelial cells. Furthermore, the peritoneal injection of ovarian cancer cells combined with exosomes derived from circWHSC1-overexpressing cells into mice promotes peritoneal dissemination. This result indicates that exosomal circWHSC1 can be transferred to peritoneal mesothelial cells, promoting peritoneal dissemination *in vivo* (Zong et al., 2019).

### Chemoresistance

Exosomal circRNAs are associated with chemotherapy resistance. A microarray analysis of exosomal RNAs from chemoresistant and chemosensitive colorectal cancer cells revealed that 105 circRNAs were significantly upregulated and 34 were downregulated in exosomes from chemoresistant cells. Interestingly, hsa_circ_0032883 was significantly upregulated in serum exosomes of chemosensitive patients compared to chemoresistant patients, suggesting that hsa_circ_0032883 may be a potential biomarker for the response to chemotherapy (Hon et al., 2019). Another study showed that ciRS-122 was positively correlated with chemoresistance in colorectal cancer. ciRS-122 acts as a miR-122 sponge and upregulates PKM2. ciRS-122 is transported from chemoresistant cells to recipient cells, where it promotes glycolysis and reduces drug susceptibility (Wang X. et al., 2020).

### Adipocyte Browning

In addition to intercellular communication between tumor cells, circRNA can be delivered into adipocytes via exosomes, promoting cancer cachexia. Cancer cachexia, which is defined as the involuntary loss of overall body weight, is associated with a poor prognosis and lower quality of life compared to cancer without cachexia. Adipocyte browning has been reported to promote cancer cachexia by aberrantly increasing thermogenesis (Kir et al., 2014; Petruzzelli et al., 2014). ciRS-133 is highly abundant in serum exosomes derived from gastric cancer patients compared with that from healthy controls. Exosomal ciRS-133 is delivered into preadipocytes, promoting their differentiation into brown-like cells by acting as a miR-133 sponge (Zhang et al., 2019b). This result indicates that tumor-derived exosomal circRNA may promote cancer cachexia.

## Potential Clinical Applications of Exosomal circRNA

circRNAs are stable due to their tertiary structure and can be detected in serum (Li et al., 2015), urine (Vo et al., 2019), and saliva (Bahn et al., 2015). Moreover, many circRNAs are dysregulated in various cancers (Naeli et al., 2020). Therefore, circRNAs appear to be promising non-invasive biomarkers for cancer diagnosis. Recent studies using high-throughput sequencing have revealed that many exosomal circRNAs serve as potential diagnostic biomarkers for cancers. Microarray analysis revealed that circ-0051443 is significantly lower in plasma-derived exosomes of patients with HCC than in healthy controls. The diagnostic performance of exosomal circ-0051443 was examined using plasma exosomes from 60 HCC patients and 60 controls. Exosomal circ-0051443 showed moderate accuracy, with an area under the curve (AUC) value of 0.8089 (Chen W. et al., 2020). In plasma exosomes from gastric cancer patients, hsa_circ_0065149, circ-KIAA, and hsa_circ_0000419 were downregulated compared to healthy controls. These circRNAs showed moderate diagnostic performance, with AUC values of 0.64, 0.75, and 0.84, respectively (Tang et al., 2018; Shao et al., 2020; Tao et al., 2020). A circRNA microarray analysis of three pairs of esophageal squamous cell cancer (ESCC) and non-tumor tissues showed that 1045 circRNAs were upregulated and 1032 were downregulated in ESCC. The expression levels of hsa_circ_0001946 and hsa_circ_0043603 in plasma from 50 ESCC patients and 50 controls were examined, and both circRNAs were significantly downregulated in the plasma of ESCC patients. Combined use of the two circRNAs showed good diagnostic performance, with 84% sensitivity and 98% specificity (Fan et al., 2019). Circulating levels of exosomal hsa-circ-0004771 were determined in serum samples from 110 colorectal cancer (CRC) patients, 35 patients with benign intestinal disease, and 35 healthy controls, and was significantly upregulated in CRC patients. Circulating exosomal has-circ-0004771 showed moderate diagnostic performance, with AUC values of 0.86 and 0.88 for differentiating stage I/II CRC patients and all CRC patients from healthy controls, respectively (Pan et al., 2019). In plasma exosomes from lung squamous cell carcinoma (LUSC) patients, RNA-Seq analysis revealed that a total of 252 circRNAs were differentially expressed compared to healthy controls. Among them, the expression levels of hsa_circ_0014235 and hsa_circ_0025580 in plasma exosomes from 30 LUSC patients and 30 controls were examined. These circRNAs showed moderate diagnostic performance, with AUC values of 0.82 and 0.80, respectively (Wang Y. et al., 2020). In lung adenocarcinoma, hsa_circRNA_0056616 was downregulated in plasma exosome. When a receiver operating characteristic curve for exosomal hsa_circRNA_0056616 levels in the context of diagnosis of lymph node metastasis was generated (*n* = 90), the AUC value was 0.812, suggesting that hsa_circRNA_0056616 could be a potential biomarker for lymph node metastasis (He et al., 2020). In small cell lung cancer, serum exosomal FECR1 was associated with poor survival and clinical response to chemotherapy (Li et al., 2019). In pancreatic cancer, circPDAC was upregulated and could be detected in patient sera (sensitivity 0.45, specificity 0.90). Interestingly, circPDAC was also detected in sera from patients with intraductal papillary mucinous neoplasm, which is a known precancerous lesion (Seimiya et al., 2020). Other exosomal circRNAs that could be potential biomarkers are summarized in Table 1.

Recently, exosomes have been explored as a therapeutic tool for the delivery of drugs to specific organs because they can evade host immune clearance and can be taken up by specific organs (Hoshino et al., 2015; Wortzel et al., 2019). For example, engineered exosomes carrying siRNA targeting the mutant oncogene KRAS inhibited advanced metastatic disease and significantly increased overall survival in mouse models of pancreatic cancer (Kamerkar et al., 2017). Recent studies have shown that exogenous circRNAs can serves as templates for protein translation, bypassing cellular RNA sensors such as RIG-I and Toll-like receptor that enable stable protein translation (Wesselhoeft et al., 2018, 2019). However, another study showed that transfection with exogenous circRNAs activated innate immunity and that m6A modification could abrogate immune activation (Chen et al., 2017; Chen Y.G. et al., 2019). Although further study is needed to determine whether circRNAs confer immunogenicity, exosomal delivery of tumor-suppressing circRNAs or circRNAs encoding therapeutic proteins may be novel cancer therapies.

## Future Perspectives

Recent research into exosomal circRNAs has revealed their abundance and diverse contributions to cancer progression. However, many questions remain unexplored. First, the regulatory mechanisms of exosomal circRNA abundance are not fully understood. The stability of circRNA and the miRNA levels associated with circRNAs in cells are likely related to the amount of circRNAs present in exosomes. Additional studies are needed to determine whether circRNAs are actively transported or passively diffuse into exosomes. Secondly, although many studies have shown that exosomal circRNAs retain biological activity as miRNA sponges, whether they can act as protein scaffolds or templates for protein translation remains unknown. Finally, studies with larger cohorts are warranted to prove that exosomal circRNAs are clinically applicable biomarkers.

## Author Contributions

TSe, MO, ET, and KK wrote the manuscript. TI, CS, and TSu prepared the figure. All authors contributed to the article and approved the submitted version.

## Conflict of Interest

The authors declare that the research was conducted in the absence of any commercial or financial relationships that could be construed as a potential conflict of interest.

## Abbreviations

AUC: area under the curve
circRNA: circular RNA
CRC: colorectal cancer
EMT: epithelial–mesenchymal transition
ESCC: esophageal squamous cell cancer
HCC: hepatocellular carcinoma
HUVECs: human umbilical vein endothelial cells
LUSC: lung squamous cell carcinoma
UCB: urothelial carcinoma of the bladder.
